# An Epigenetic Switch Involving Overlapping Fur and DNA Methylation Optimizes Expression of a Type VI Secretion Gene Cluster

**DOI:** 10.1371/journal.pgen.1002205

**Published:** 2011-07-28

**Authors:** Yannick R. Brunet, Christophe S. Bernard, Marthe Gavioli, Roland Lloubès, Eric Cascales

**Affiliations:** Laboratoire d'Ingénierie des Systèmes Macromoléculaires, CNRS – UPR 9027, Institut de Microbiologie de la Méditerranée, Aix-Marseille Université, Marseille, France; Uppsala University, Sweden

## Abstract

Type VI secretion systems (T6SS) are macromolecular machines of the cell envelope of Gram-negative bacteria responsible for bacterial killing and/or virulence towards different host cells. Here, we characterized the regulatory mechanism underlying expression of the enteroagregative *Escherichia coli sci1* T6SS gene cluster. We identified Fur as the main regulator of the *sci1* cluster. A detailed analysis of the promoter region showed the presence of three GATC motifs, which are target of the DNA adenine methylase Dam. Using a combination of reporter fusion, gel shift, and *in vivo* and *in vitro* Dam methylation assays, we dissected the regulatory role of Fur and Dam-dependent methylation. We showed that the *sci1* gene cluster expression is under the control of an epigenetic switch depending on methylation: fur binding prevents methylation of a GATC motif, whereas methylation at this specific site decreases the affinity of Fur for its binding box. A model is proposed in which the *sci1* promoter is regulated by iron availability, adenine methylation, and DNA replication.

## Author Summary

DNA methylation plays an important role in the regulation of genes involved in assembly of cell surface adhesins or appendages. Methylation at a GATC motif by the Dam methylase influences binding of transcriptional regulators, leading to variation in the gene expression pattern. In several cases, this may lead to different cell subpopulations allowing a rapid adaptation to varying environments. In this work, we uncover the regulatory mechanism controlling expression of the *sci1* Type VI secretion gene cluster in entero-aggregative *Escherichia coli*, which encodes a structure required for inter-bacterial interaction. We showed that this gene cluster is repressed by Fur in iron-replete conditions and that Fur binding on the promoter prevents methylation of a GATC motif. In iron-limited conditions, Fur is relieved from the promoter allowing expression of the gene cluster and methylation of the GATC motif. Methylation prevents de novo Fur binding allowing constitutive expression. Our findings support a model in which the expression of the Type VI secretion gene cluster is regulated by a non-stochastic epigenetic switch: switch from the OFF to ON phases depends on iron availability whereas the ON to OFF switch depends on DNA replication and competition between Dam-dependent methylation and Fur binding.

## Introduction

Type VI secretion systems (T6SS) are macromolecular assemblies encoded within the genome of most Gram negative bacteria [1]–[3]. They are composed of at least 13 subunits, called core components, which are believed to form a trans-envelope apparatus from the cytoplasm to the outside of the cell [2]. Several subunits of this transport system have extensive homologies with Type IV secretion components, or functional homologues in envelope spanning complexes such as an outer membrane lipoprotein [4], a protein anchoring the system to the peptidoglycan layer [5], [6] and an AAA+ ATPase. The exciting discoveries that two core components exhibit remarkable structure conservation with two bacteriophage structural proteins reshaped this field [7]. Two T6SS proteins released in the environmental milieu, Hcp and VgrG, are structurally related to the tail tube and the cell-puncturing device of bacteriophage T4, gp19 and the gp27-gp5 complex respectively [8]–[11]. From these data, it has been suggested that T6SS will assemble a bacteriophage upside-down structure, anchored to the cell envelope through the bacteriophage-unrelated membrane or membrane-associated subunits. In this model, Hcp will assemble a tube-like structure resembling the bacteriophage tail, and displaying a VgrG trimer at the tip [7], [10]. Interestingly, a number of VgrG proteins are fused to an additional, C-terminal domain, carrying an effector function [12]. It has been initially proposed that T6SS are important virulence factors towards eukaryotic host cells [13]; however, although this turned to be true in several cases, most VgrG proteins do not carry the C-terminal extension.

Recently, several research groups demonstrated that a number of T6SS, including those of *Pseudomonas aeruginosa*, *Burkholderia thailadensis* and *Vibrio cholerae* are required for inter-bacterial competition, and anti-bacterial toxins secreted by the *P. aeruginosa* HSI-1 T6SS have been identified [14]–[17]. Indeed, mixed cultures between a T6SS-producing strain and a different species showed killing of the T6SS non-producing in a T6SS-dependent manner. Therefore, the role of T6SS in host pathogenesis is somehow limited to the competition towards other microorganisms, to gain access to a specific niche where additional virulence factors may act directly against host cells [16], [18]. However, the roles of T6SS are not limited to virulence towards host cells or towards surrounding bacteria, but several studies reported roles in resisting amoeba predation, stress sensing, or biofilm formation [4], [13], [19]. It appears that T6SS are adapted to the specific needs of each individual bacterium, and are therefore subjected to specific and precise regulatory modulations [20], [21]. Indeed, a wide array of different mechanisms have been reported: control by quorum sensing mechanisms, two-component systems, transcriptional factors, histone-like proteins or alternate sigma factors [20]–[22].

In this study, we sought to identify the regulatory mechanism underlying expression of the enteroaggregative *Escherichia coli* (EAEC) *sci1* T6SS gene cluster. Using random mini-*Tn* mutagenesis of a strain carrying a translational reporter fusion to the *sci1* promoter, we identified the Ferric uptake regulator Fur as the main repressor of the expression of this cluster. The Fur protein has been well characterized in several bacteria in which it acts as a transcriptional repressor of iron-regulated promoters [23]. In presence of iron, Fur represses the expression of these promoters, while in absence of iron, Fur is relieved from these promoters leaving access for the RNA polymerase [23]. The target promoters of Fur have been identified in *E. coli* K12 and a consensus Fur binding sequence (or Fur box) has emerged [23]. We identified two Fur boxes in the promoter region of the *sci1* T6SS gene cluster, including one overlapping with the putative -10 box. The direct binding of Fur was further confirmed by *in vivo* Fur titration and *in vitro* gel shift assays. Interestingly, close analysis of the promoter elements and Fur binding boxes showed an overrepresentation of GATC motifs, which are targets for the DNA adenine methylase Dam. Dam catalyzes methylation at the N^6^ position of the adenine of the GATC motif. Dam methylation has been shown to be involved in a variety of processes, including the control of the timing of replication, mismatch repair or transcriptional regulation [24]–[27]. In several cases, Dam-dependent methylation modulates DNA-protein interactions [28]). The genes under the control of this mechanism display a phase variation expression pattern, in which binding of the transcriptional factor depends of the methylated state of the DNA [29]. Using a combination of *in vivo* and *in vitro* methylation assays, as well as *in vitro* Fur binding assays on nonmethylated and methylated *sci1* promoter, we demonstrated that the *sci1* promoter expression depends on the outcome of the competition between Fur binding and Dam-dependent methylation. We propose that the *sci1* gene cluster expression undergoes an epigenetic switch, varying from an ON to an OFF state in response to iron availability and DNA replication.

## Results

### The *sci1* T6SS gene cluster is weakly expressed in *in vitro* conditions

The first gene of the enteroaggregative *E. coli* (EAEC) *sci1* T6SS gene cluster, *sciH*, is preceded by a 578-bp non-coding sequence, which is hereafter called *sci1* promoter. To test the activity of the *sci1* promoter, we constructed a *lacZ* transcriptional reporter fusion in which *lacZ* expression is controlled by the *sci1* promoter region (from –578 to + 18 [relative to the *sciH* start codon]). The 596-pb DNA fragment corresponding to the *sci1* promoter region has been inserted into the *Sma*I site of the pBR322-derived multicopy plasmid pGE593 [30], encoding a promoterless *lacZ* gene. Pilot studies performed in LB medium showed that the basal level of β-galactosidase produced from the promoterless *lacZ* gene was quite high (∼ 180 Miller units), whereas the *sci1*-*lacZ* fusion only displayed a 3- to 4-fold higher expression (∼600–800 Miller units). To avoid any artifactual effect on fusion activity due to the high copy number of the plasmid, we deleted the chromosomal *pcnB* gene, a gene involved in the regulation of the copy number of pBR322-derived vectors [31]. In this background, the activity of the promoterless fusion was considerably decreased (∼ 40 Miller units). We also noted that the *sci1-lacZ* fusion was poorly expressed in LB rich medium. We then tested the activity of the *sci1*-*lacZ* fusion under several conditions. We found that the activity of the *sci1*-*lacZ* fusion increased in the late stage of exponential growth phase and in stationary growth phase, after acid exposure or in minimal media (data not shown).

### Random mini-*Tn*10 transposon mutagenesis identified Fur as a regulator of EAEC *sci1* expression

**Figure 1:**
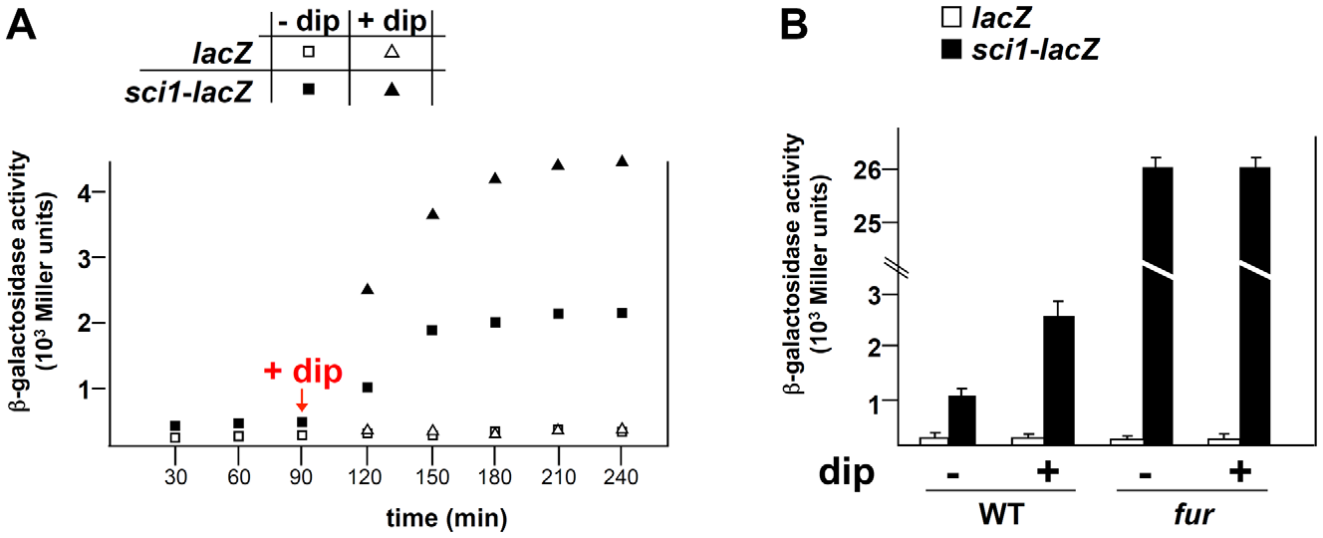
The EAEC *sci1* T6SS gene cluster is regulated by iron levels and the Fur repressor. *(A)* β-galactosidase activity of a promoterless *lacZ* fusion (open symbols) and of the *sci1-lacZ* reporter fusion (closed symbols) upon addition of the iron chelator 2,2′-dipyridyl (dip; 100 µM, squares) in a EAEC wild-type (WT) strain (triangles: no dip added). *(B)* β-galactosidase activity of a promoterless *lacZ* fusion (white bars) and of the *sci1-lacZ* reporter fusion (black bars) after 120 minutes of culture (OD_600nm_ = 0.8) upon a 30 min treatment with 2,2′-dipyridyl (+dip; 100 µM) or ethanol-carrier (-dip) in a WT strain or its isogenic *fur* mutant.

To gain further insight onto the *sci1* regulatory mechanism, we performed a transposon mutagenesis to identify regulators. Cells carrying the *sci1-lacZ* fusion, which form white to light blue colonies on X-Gal LB (pH 8.0) plates, were transformed with the pNKBOR plasposon, a suicide vector carrying a mini-Tn*10* transposon and its cognate transposase [32]. We screened ∼20,000 kanamycin-resistant clones for higher *lacZ* activity and obtained three dark blue clones with a ∼25-fold increase in β-galactosidase activity (data not shown). Sequencing the site of transposition revealed that insertions occurred at three independent positions within the *fur* gene, which encodes the master regulator of iron and pH homeostasis (data not shown). Since *Tn* insertions resulted in the disruption of the *fur* gene, these data suggest that the Fur protein may act as a negative regulator of *sci1* gene cluster expression. To independently verify the role of Fur in *sci1* expression, we measured the activity of the *sci1-lacZ* reporter fusion in presence of the iron chelator 2,2′-dipyridyl. As shown in Figure 1A, the activity of the reporter fusion increased upon exposure to dipyridyl. Construction of the EAEC *fur* null strain confirmed the transposon mutagenesis data (Figure 1B). By contrast, deletion of *aggR*, the gene encoding the AraC-like transcriptional activator of the EAEC *sci2* T6SS gene cluster [33], had no effect on the promoterless and the *sci1-lacZ* fusions (data not shown).

### Sequence analysis of the *sci1* promoter region

**Figure 2:**
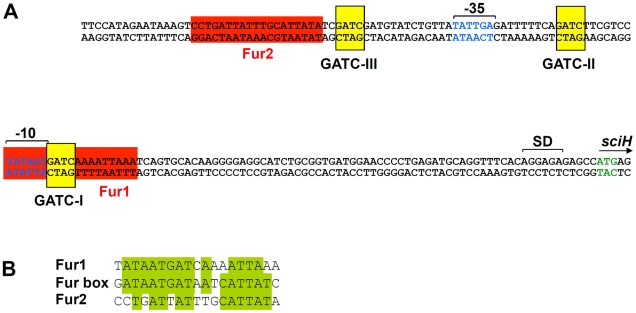
*In silico a*nalysis of the *sci1* proximal promoter region. *(A)* The proximal *sci1* promoter region. The ATG translational codon of *sciH* is indicated as well as the Shine Delgarno (SD). The putative -10 and -35 elements of the σ^70^ promoter (identified by the BProm algorithm) are indicated in blue, as well as Fur-binding sequences (red boxes) and GATC Dam-dependent methylation sites (yellow boxes). The two Fur-binding sequences and GATC sites are numbered from the start site. *(B)* Sequence alignment of the *fur1* and *fur2 sci1* boxes with the *E. coli* Fur box consensus sequence. Identical bases are framed in green.

The Fur protein has been extensively studied. Fur acts as a dimer and participates in regulation of genes involved in iron homeostasis and tolerance to acid stresses [23]. Once complexed to iron, Fur binds to a well-defined 19-bp sequence (GATAATGATAATCATTATC), called the ‘Fur box’ [23]. To determine whether the effect of the *fur* mutation was direct, we first analyzed the *sci1* promoter sequence. Interestingly, two putative Fur binding sites were identified (Figure 2A). The *fur2* binding box (11 out of the 19 nucleotides of the consensus Fur box, see Figure 2B) is located upstream the putative -35 element of the putative σ^70^ promoter. *fur1* (13 out of the 19 nucleotides of the consensus Fur box, see Figure 2B) overlaps with the putative -10 box. The position of the *fur1* box suggests that Fur may prevent RNA polymerase (RNAP) binding to the -10 element, a characteristic commonly observed for transcriptional repressors, including the Fur protein [34], [35]. Overall, the *in silico* analysis of the *sci1* promoter sequence suggests that Fur directly bind to the *sci1* promoter.

### The Fur protein binds to the *sci1* promoter at *fur1* and *fur2*

**Figure 3:**
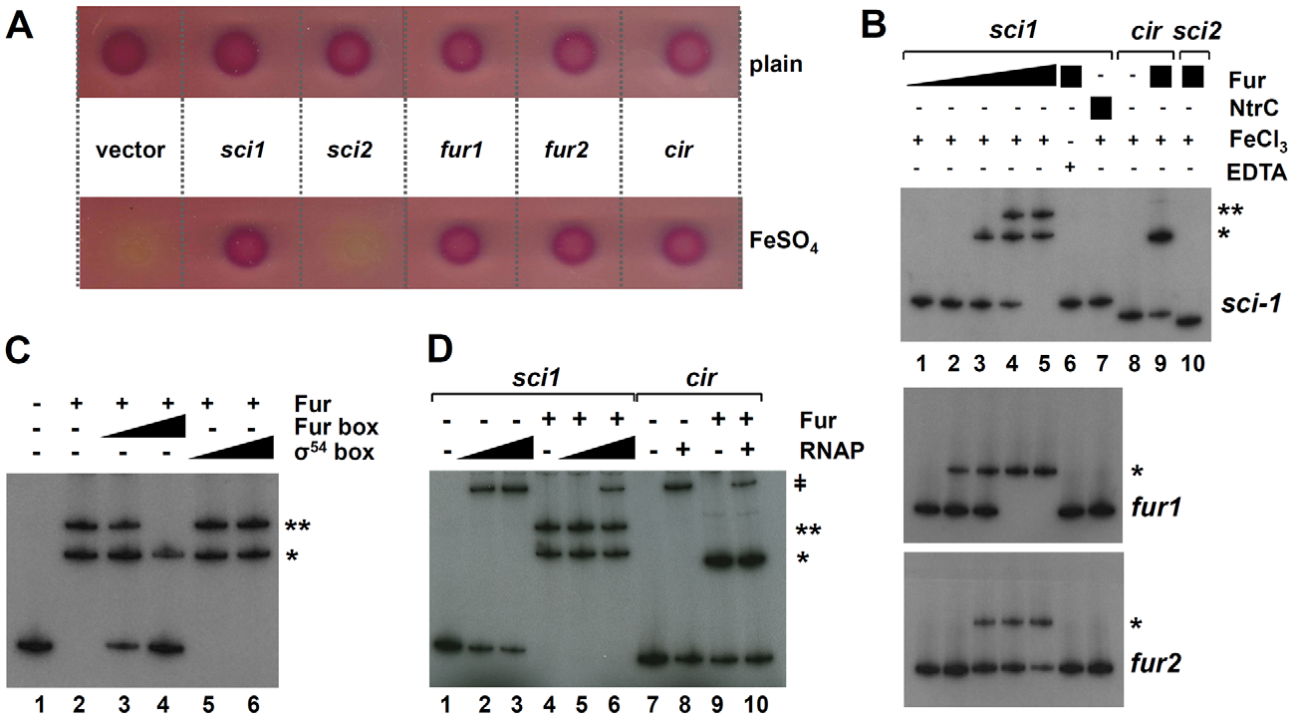
Fur binds to the EAEC *sci1* T6SS promoter *in vivo* and *in vitro.* * (A)* Fur Titration assay (FURTA). H1717 reporter cells (*fhuF-lacZ*) carrying the empty vector or the vector bearing the *sci1*, *sci2*, or *cir* promoters, or the *fur1* or *fur2* sequences were spotted on MacConkey plates (upper panel) or on MacConkey plates supplemented with FeSO4 (30 µM; lower panel). A *lacZ+* phenotype reports a derepression of the *fhuF-lacZ* reporter fusion by titration of the Fur protein bound to the *fhuF* promoter. *(B)* Electrophoretic mobility shift assay of the *sci1* promoter (upper panel) or of the *fur1* (middle panel) or *fur2* (lower panel) sequences using purified Fur (lane 1, no protein; lane 2, 0.5 nM; lane 3, 2 nM, lane 4, 5 nM, lane 5, 20 nM) in presence of FeCl3 or in presence of EDTA (lane 6, Fur at 20 nM) or using purified NtrC transcriptional activator (lane 7, 50 nM). Controls include Fur shift assays of the Fur-dependent *cir* promoter (lane 8, no protein; lane 9, Fur at 5 nM) or of the Fur-independent *sci2* promoter (lane 10, 20 nM). *(C)* Competition experiments for Fur binding (lane 1, no protein; lanes 2–6, Fur at 20 nM) with duplex consensus Fur- (lane 3, molecular ratio *sci1*:*fur box* 1∶2; lane 4, molecular ratio 1∶10) or σ54-binding sequence (lane 5, molecular ratio *sci1*:σ^54^-box 1∶2; lane 6, molecular ratio 1∶10). *(D)* Binding of the Eσ^70^ RNA polymerase holoenzyme (RNAP) (lanes 1, 4, 7 and 9, no RNAP; lanes 2 and 5, RNAP 0.5 unit; lanes 3, 6, 8 and 10, RNAP 2 units) on the *sci1* or control *cir* promoter pre-incubated (+) or not (−) with Fur (20 nM). Fur-DNA, (Fur)_2_-DNA, and RNAP-DNA complexes are indicated by *, **, and ǂ respectively.

To test whether Fur binds the *sci1* promoter *in vivo*, we used the Fur titration assay (FURTA [36]). In this assay, a chromosomal *fhuF::lacZ* fusion is derepressed if a Fur box is carried on a high copy plasmid. We thus cloned the *sci1* promoter sequence as well as the two putative Fur boxes (the *fur1* and *fur2* sequences flanked by the natural downstream and upstream 3 bases) and controls (the Fur-dependent *cir* and Fur-independent *sci2* promoters) into the high copy pT7.5 vector. Figure 3A shows that transformation of the reporter strain with pT7.5 derivatives carrying the *sci1* promoter or of the two putative Fur boxes derepressed the *fhuF::lacZ* fusion, leading to a *lac* ^+^ phenotype on MacConkey agar plates supplemented with FeSO_4_.

Fur binding to the *sci1* promoter was then confirmed *in vitro*. The *E. coli* K12 Fur protein was purified to homogeneity by metal affinity chromatography and tested for its ability to bind the *sci1* promoter by electrophoretic mobility shift assays (EMSA) (Figure 3B, upper panel). As expected, the *cir* promoter fragment was shifted by the Fur protein in a metal-dependent manner, whereas no shift was observed for the *sci2* promoter fragment, even at high Fur concentration. The Fur protein bound the *sci1* promoter, in a metal-dependent manner. Interestingly, two shifts were observed, suggesting formation of two distinct complexes. Digestion of the *sci1* promoter fragment by SspI leads to two fragments, a 384-bp 5′ DNA fragment and a 212-bp 3′ DNA fragment which contains the two putative Fur boxes. Following SspI digestion and EMSA, we observed that the 212-bp fragment—but not the 396-bp fragment—was retarded in the presence of the Fur protein (data not shown). We further tested whether two fragments encompassing the *fur1* or the *fur2* box were retarded by Fur. Figure 3B shows that both fragments were retarded although the affinity of Fur was higher for the *fur1* box. Specificity of Fur binding was further confirmed by using specific (a duplex consensus 19-bp Fur box flanked by 7 bases) or non-specific (a duplex consensus 22-bp σ^54^ box flanked by 7 bases) unlabelled competitors (Figure 3C). Overall, our data suggest that Fur binds the *sci1* promoter, including at a position that overlaps with one of the RNAP-binding elements. We then tested whether Fur exerts a competitive effect to RNAP binding. As expected from the position of the *fur1* box relative to the putative -10 element, pre-incubation of the *sci1* promoter probe with Fur decreased the affinity of RNAP for the DNA fragment (Figure 3D).

### The Fur protein prevents Dam-dependent methylation at GATC-I

Interestingly, sequence analysis of the *sci1* promoter also revealed the existence of three GATC motifs over a 53-nucleotide region (see Figure 2A). The adenine of GATC motifs are recognized and methylated by the DNA adenine methylase (Dam). Two of these sites (GATC-II and GATC-III) flank the putative -35 element whereas the third site, GATC-I, is located at the 3′ of the putative -10 element and overlaps with the *fur1* box (Figure 2A).

**Figure 4:**
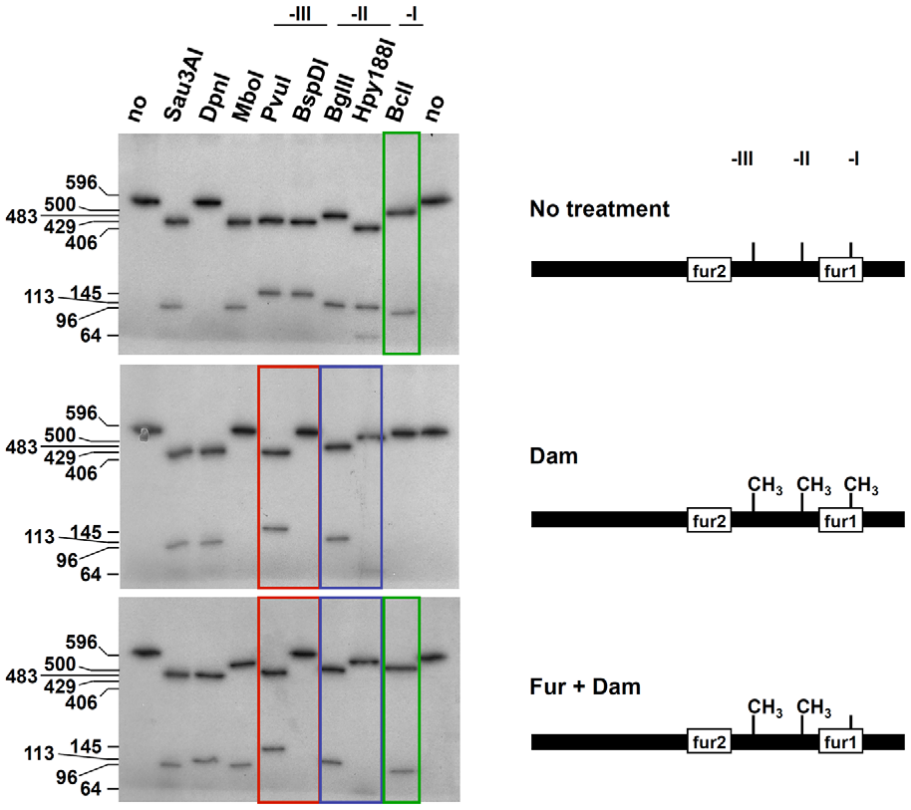
Fur protects GATC-I from methylation *in vitro*. A radiolabeled PCR product corresponding to the 596-bp *sci1* promoter was digested by the restriction enzymes indicated on top (no, no digestion). Upper panel, untreated PCR product; middle panel, PCR product treated with the Dam methylase; lower panel, PCR product incubated with purified Fur (20 nM) prior to Dam methylation. The sizes of the digestion products (in bp) are indicated on the left. Red and blue frames emphasize the observation that incubation with Fur did not change the digestion profiles for GATC-II (-II) and GATC-III (-III) whereas the green frame emphasize the observation that GATC-I (-I) was not methylated upon Fur binding. Schematic representations of the conclusions of the left panels are shown on right. See Figure S1 for positions of restriction sites and sizes of DNA fragments.

**Figure 5:**
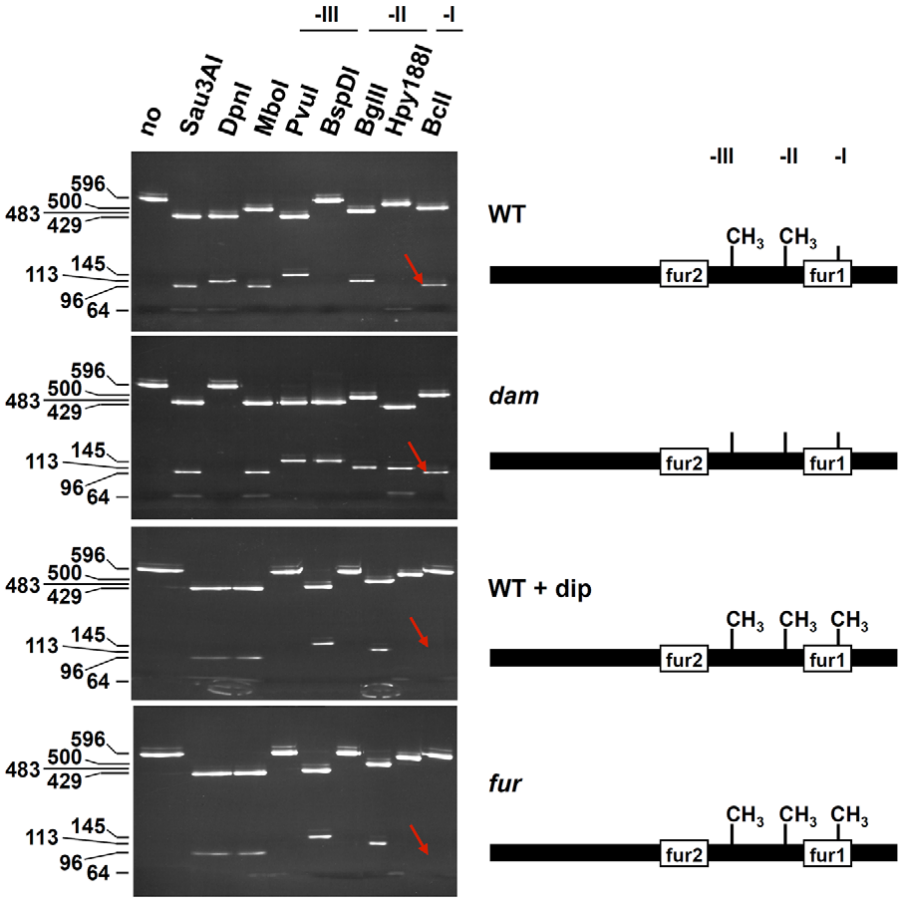
Fur protects GATC-I from methylation *in vivo*. The *sci1* promoters purified from the EAEC wild-type strain (WT, upper panel) or its isogenic *dam* (second panel rom top) or *fur* (lower panel) mutant strains, or from the WT strain treated with 2,2′-dipyridyl (WT + dip; third panel from top) were digested by the restriction enzymes indicated on top (no, no digestion). The sizes of the digestion products (in bp) are indicated on the left. Arrows indicate the position of the digestion product obtained with the BclI restriction enzyme emphasizing the observation that GATC-I was not methylated in a WT strain but was methylated in a *fur* mutant strain (or in a WT strain treated with 2,2′-dipyridyl). Schematic representations of the conclusions of the left panels are shown on right. See Figure S1 for positions of restriction sites and sizes of DNA fragments.

**Figure 6:**
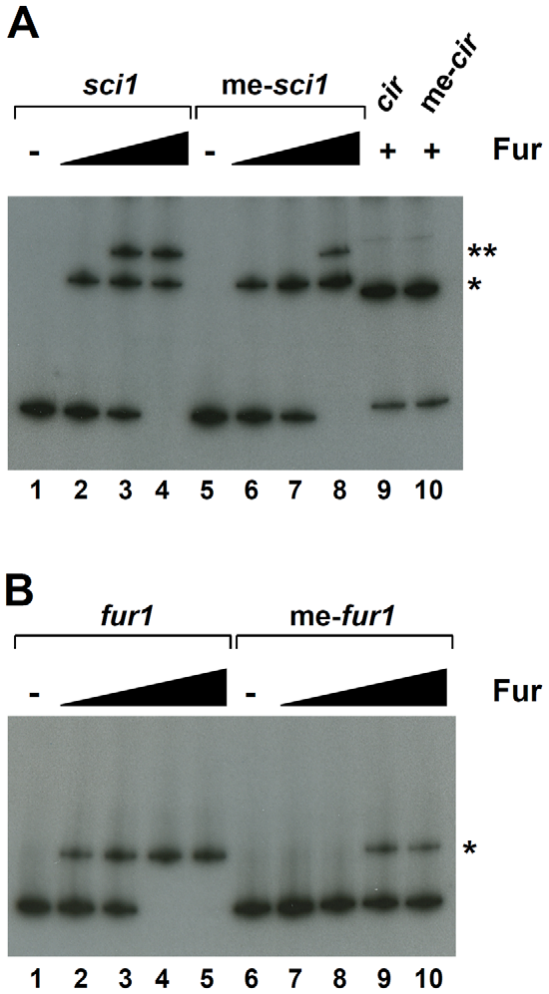
GATC methylation influences Fur binding on *fur1*. *(A)* Electrophoretic mobility shift assay of the non methylated or Dam-methylated (me-) *sci1* or *cir* promoter using purified Fur (lanes 1 and 5, no protein; lanes 2 and 6, 2 nM; lanes 3 and 7, 5 nM; lanes 4 and 8-10, 20 nM). *(B)* Electrophoretic mobility shift assay of the non methylated or Dam-methylated (me-) *fur1* sequence using purified Fur (lanes 1 and 6, no protein; lanes 2 and 7, 0.5 nM; lanes 3 and 8, 2 nM; lanes 4 and 9, 5 nM; lanes 5 and 10, 20 nM). Fur-DNA, (Fur)_2_-DNA complexes are indicated by *, and ** respectively.

The observation that the *fur1* box contains a GATC site (GATC-I) suggests that Fur and Dam-dependent methylation overlap for the regulation of the *sci1* T6SS gene cluster. Several examples have been reported of interplays between the methylation at GATC sequences and binding of transcriptional regulators, including Lrp and OxyR, as a phenomenon known as “phase variation” [24]–[29]. We therefore tested whether Fur influences Dam-dependent methylation (Figure 4 and Figure 5) and vice-versa (Figure 6). We took advantage of the observation that each GATC site within the *sci1* promoter was part of a 6-nucleotide palindrome sequence (see Figure S1) for which methylation-sensitive or –insensitive restriction endonucleases are commercially available. We then compared the digestion profile of a PCR-generated fragment encompassing the 596-bp of the *sci1* promoter. The non-methylated PCR product was digested by all restriction nucleases with the exception of the methylated GATC-specific DpnI enzyme (Figure 4, upper panel). Upon *in vitro* methylation by Dam, all the sites were methylated and thus, restriction nucleases sensitive to Dam methylation (MboI, BspD1, Hpy188I, and BclI) were inactive on this fragment (Figure 4, middle panel). When Dam was added to a mixture containing the PCR product and an excess of the Fur protein, the Dam-sensitive BspD1 and Hpy188I nucleases were inactive, demonstrating that the GATC-II and –III sites were methylated (Figure 4, lower panel). By contrast, the methylation-sensitive BclI nuclease was active on the GATC-I site, revealing that the GATC-I site remained non-methylated in presence of Fur. These results thus demonstrate than Fur protects GATC-I from methylation *in vitro*.

We then tested the methylation state of each GATC sequence of the *sci1* promoter *in vivo*. The plasmid carrying the *sci1-lacZ* fusion was extracted from various genetic backgrounds and each GATC site was analyzed using the restriction assay. In the *dam* derivative, none of the sites was methylated (Figure 5, second panel from top). Interestingly, the GATC-II and –III sites were methylated in a wild-type background as shown by the absence of activity of the methylation-sensitive BspDI and Hpy188I nucleases on the promoter substrate (Figure 5, top panel). However, GATC-I remained nonmethylated, suggesting it is protected from Dam-dependent methylation *in vivo*. This protection was due to the presence of the Fur protein bound to the *fur1* box since the GATC-I site was fully methylated in *fur* mutant cells (Figure 5, lower panel) or when wild-type cells were exposed to 2,2′-dipyridyl (Figure 5, third panel from top). Overall, the results showed in Figure 4 and Figure 5 demonstrate that Fur binding prevents DNA adenine methylation at the GATC-I site.

### GATC-I methylation decreases affinity of Fur for the *fur1* binding site

Reciprocally, we tested the effect of DNA adenine methylation on Fur binding. A radiolabelled PCR product was methylated *in vitro* by Dam, and then used as probe in electrophoretic mobility shift assays. As shown in Figure 6, gel shift assays demonstrated that methylation of the *sci1* promoter fragment decreased the affinity of Fur whereas had no effect on the control *cir* promoter (Figure 6A). However, only one of the two Fur binding boxes seemed affected since methylation of the *sci1* promoter affected the formation of the (Fur)_2_-DNA complex. We therefore tested whether methylation of the *fur1* box affected Fur binding. As shown in panel *(B)*, methylation of the *fur1* box had a negative impact on Fur binding (Figure 6B).

## Discussion

**Figure 7:**
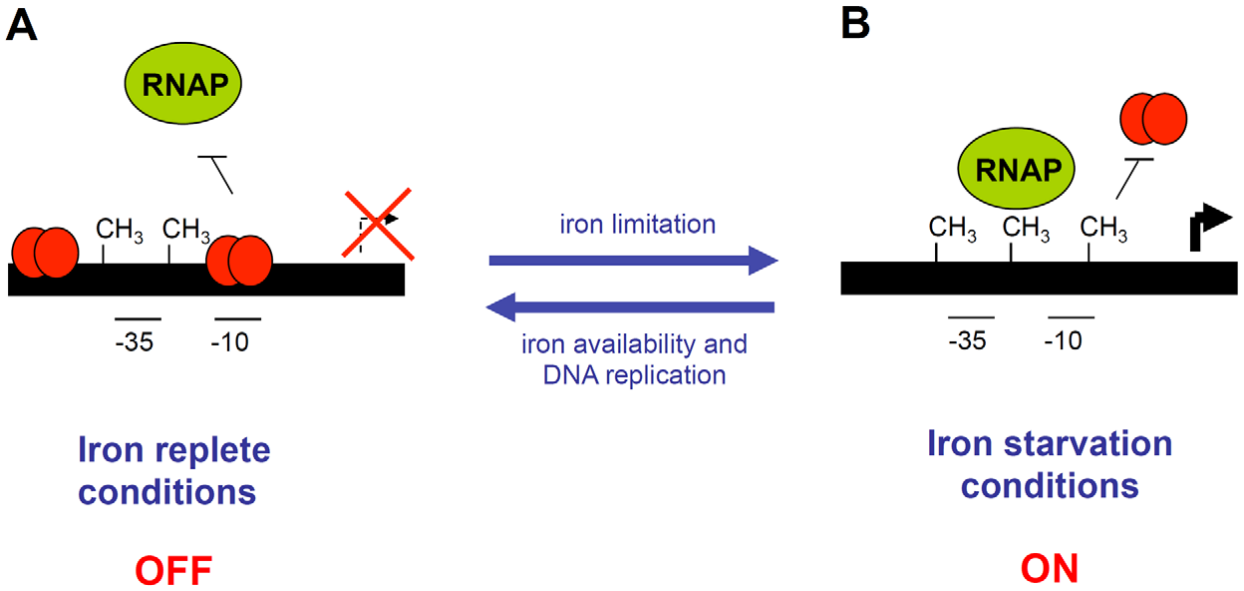
Schematic representation of the EAEC *sci1* T6SS gene cluster epigenetic switch regulatory mechanism. *(A)* In iron-replete conditions, Fur (red balls) represses the expression of the *sci1* gene cluster by binding to specific boxes overlapping the putative -10 transcriptional element. The expression of the *sci1* gene cluster is in the OFF phase. *(B)* In iron starvation conditions, Fur is relieved from the putative -10 element, leaving the promoter available for RNAP binding and transcription. Dam-dependent methylation at the GATC-I site prevents Fur binding. The expression of the *sci1* gene cluster is in the ON phase. Transition to the OFF phase requires both iron replete conditions and hemi-methylation of GATC-I after DNA replication. Methylated GATC sites are indicated by CH_3_.

In this study, we have shown that the *sci1* Type VI secretion gene cluster is poorly expressed under the laboratory conditions. Using a combination of random mutagenesis, reporter fusion, titration and gel mobility shift assays, we have shown that Fur represses the expression of this cluster. We also demonstrated that expression of this cluster is modulated by DNA adenine methylation at the GATC-I site by the Dam methylase. Using *in vivo* and *in vitro* methylation protection assays, we showed that Fur binding prevents methylation at the GATC-I site, whereas methylation at GATC-I decreases affinity of Fur for its binding sequence (see Figure 7 and below).

### Fur regulation

Although suggested for the regulation of the *Edwardsiella tarda* Evp and the distantly-related *Francisella tularensis* FPI Type VI secretion systems, the role of the Fur protein has not yet been characterized in the regulation of these clusters [20]. In the case of the *sci1* gene cluster, we identified two Fur boxes, including one—the *fur1* box—overlapping with the putative -10 box of the putative σ^70^-dependent promoter. Indeed, using competition gel shift experiments we have shown that Fur prevents RNA polymerase binding at the *sci1* promoter. The *fur2* box being located upstream the promoter, its role is not yet clear, but one may hypothesize that this second, lower affinity, box has a role in cooperativity, *i.e.*, increasing the local concentration of the Fur protein around the *sci1* promoter. Further experiments are therefore required to clearly understand the specific role of each Fur binding box.

### Dam regulation

We also observed a role of Dam methylation in the regulation of the EAEC *sci1* gene cluster. Interestingly, Dam methylation site are over-represented in the *sci1* promoter: whereas a GATC sequence should statistically be present every ∼250 bases, three GATC sequences are present within a 53-nucleotide sequence flanking the putative -35 and -10 elements of the promoter. This represents a 10-fold increase over random average. Dam methylation has been shown to be involved in a variety of processes such as mismatch repair, temporal regulation of the replication, as well as transcriptional regulation [25], [28]. In this latter case, it is noteworthy that most Dam-dependent gene regulations have been identified in the case of genes encoding cell surface structures such as conjugation machines, Type III secretion systems, several pili and fimbriae, adhesines, or enzymes required for the modification of the antigen O of the lipopolysaccharide [25], [37]. In several of these cases, methylation may cause biphase or phase variation, allowing that bacteria produce variable structures at the cell surface and to escape host immunogenicity. Although T6SS-dependent structures have not been yet observed at the cell surface, the presence of both Hcp and VgrG in cell culture supernatant and the homologies of these proteins with tail tube and syringe components of the bacteriophage T4 suggest that these proteins might form an extracellular appendice. It is noteworthy that if this were the case, the regulation of this cell surface structure will be dependent on Dam, which has been previously shown to be involved in the regulation of various pili, fimbriae or adhesines. More striking, these cell surface structures are involved in adhesion or biofilm formation such as the EAEC *sci1* Type VI secretion system [4].

### Fur and Dam interplay

The most interesting data generated in this study concerns the competitive effect of the Fur and Dam proteins at the -10 box. Using *in vivo* and *in vitro* methylation protection assays, we found that Fur binding prevents methylation at the GATC-I site. We observed that GATC-I was unmethylated in the WT strain, whereas became methylated upon exposure of this strain to 2,2′-dipyridyl or in a *fur* mutant strain. It is noteworthy that these experiments were done using a low copy plasmid, and that it remains to test whether identical methylation patterns occur on the chromosome. Methylation of a GATC site depending on the presence of a regulatory protein has been exemplified in several cases (see [25]) such as the regulation of the *gut*, *carAB* or *agn43* operons, in which binding of the GutR, CarP or OxyR transcriptional factors on the promoter prevents Dam methylation [38]–[40]. In reciprocal experiments, using gel mobility shift assays, we showed that Fur had a lower affinity for the *fur1* box upon GATC-I methylation. Therefore, the addition of a methyl group on the adenine of the GATC-I motif is sufficient to diminish the Fur affinity for the *fur1* box. This nucleotide is conserved in the consensus Fur binding motif (see Figure 2B) and the adjacent thymine residue is directly engaged in interaction with the repressor [23]. Consistent with these findings, Dam-dependent methylation abrogating binding of transcriptional activators had been reported [41], [42]. It has been suggested that methylation interferes with transcriptional factors binding by direct steric occlusion or by the local modification in the DNA conformation [28].

From these data, we propose that the expression of the *sci1* Type VI secretion gene cluster is under the control of a regulatory mechanism controlling transitions between ON and OFF expression states (Figure 7). In this model, the switch will be controlled by the level of iron and by an epigenetic mechanism involving the Dam methylase. In iron rich conditions, Fur will prevent RNAP binding at the -10 box, as well as GATC-I site methylation: the expression of the *sci1* gene cluster will be stably maintained in a repressed state (OFF phase). To switch to the ON state, Fur must be displaced from the *fur1* box. In iron-limited conditions, Fur will be relieved from the promoter, allowing expression of the gene cluster. Fur displacement will leave the GATC-I site available for Dam-dependent methylation, which will in turn prevent *de novo* Fur binding, allowing the expression of the *sci1* gene cluster to be maintained in a stable ON phase. Dam thereby acts as a positive regulator by stabilizing the ON expression state. In this model, *fur* mutant cells are locked in the ON state, whereas *dam* mutant cells are locked in the OFF phase, which is consistent with our reporter fusion studies.

Although different, this elegant mechanism resembles phase variation, the better studied examples being the regulation of *agn43* which involves the OxyR repressor and Dam methylation [40]–[43], and the Pap (pyelonephritis-associated pili) switch, which involves the Leucine-responsive protein Lrp and Dam methylation (for a review, see [44]). In both cases, competition between the transcriptional factor and Dam methylation allows the transition between the OFF and ON transcriptional states [25]. In phase variation mechanisms, the stochastic passage from the ON to the OFF phase leads to the formation of subpopulations. In the mechanism described in this study, the expression of the *sci1* T6SS is controlled by iron levels and hence, no subpopulations have been observed (data not shown). Although epigenetic switches resulting from competition between Dam methylation and transcriptional activators have been reported, this is the first study demonstrating competitive effects between Dam and the Fur repressor. It is noteworthy that no GATC site is found in the putative Fur-binding boxes of the *E. tarda* or *F. tularensis* T6SS gene clusters, suggesting that the Fur/Dam interplay mechanism is not widely distributed for the expression of T6SS gene clusters.

### A model of the *sci1* epigenetic switch

In the case of the Pap switch, it has been shown that transition to the ON phase requires the PapI protein, the expression of which is induced by PapB. The OFF-ON transition is therefore controlled by PapB and PapI. It has been proposed that this modulation is a stochastic event, although environmental factors may somehow influence this modulation [44]. In the case of the *sci1* promoter, the passage of the OFF to the ON phase is probably less random, and is essentially dependent upon the iron availability; however maintenance in a stable ON phase requires methylation of the GATC-I site. How the bacteria manage the passage from the ON to the OFF phase is an interesting question. The ON to OFF switch requires a change from a methylated to nonmethylated state of the GATC-I sequence, which can only occurs upon DNA replication. In the case of *agn43*, it has been shown that OxyR can bind to the hemimethylated operator upon DNA replication [45]. However, it is noteworthy that GATC-I methylation decreases Fur affinity for *fur1* (and does not abrogate it). It remains possible that Fur binds to a hemimethylated *fur1* box, therefore facilitating the ON to OFF switch during DNA replication in iron replete conditions. In this model, the absence of DNA replication should maintain the expression of the *sci1* gene cluster in an ON state, irrespective of iron levels, a hypothesis that remains to be tested. Further studies using quantitative competition experiments between Fur and Dam methylation, as well as Fur binding on hemimethylated templates will probably provide important details on this mechanism. In sum, we suggest that Dam methylation will therefore serve two roles: (i) preventing *de novo* Fur binding in absence of DNA replication (irrespective of iron levels), and (ii) slowing down *de novo* Fur binding upon DNA replication. This mechanism will timely regulate expression of the *sci1* T6SS gene cluster.

On a physiological perspective, it is difficult to reconcile the regulatory mechanism dissected in this study and the role of the EAEC T6SS in the timing of biofilm formation. One may hypothesize that iron limitation occurs in the digestive track due to high competition between micro-organisms or by the activation of host mechanisms to sequester iron at the mucosal surface [46]. Several regulatory mechanisms have thus been developed by pathogens to induce virulence genes expression in response to iron starvation [46]. Iron starvation may thus have been hijacked by EAEC to initiate the switch of the population to the ON phase, and thus to timely regulate biofilm formation.

## Materials and Methods

### Bacterial strains, media, growth condition, and chemicals

Strains used in this study and their relevant characteristics are listed in Table S1. *Escherichia coli* K12 DH5α was used for cloning procedures. The enteroaggregative *E. coli* strain 17-2 (kindly provided by Arlette Darfeuille-Michaud, University of Clermont-Ferrand, France) was used for this study. The FURTA reporter strain (H1717, *fhuF*::*lacZ* [36]) was generously provided by Klaus Hantke (Tuebingen Universitat, Germany). The *E. coli* K12 BW25113*fur*Ω*kan* and *dam*Ω*kan* strains from the KEIO collection [47] were obtained through Patrice L. Moreau (LCB, Marseille). Strains were routinely grown in LB broth at 37°C, with aeration. When required, M9 minimal medium supplemented with glucose 0.4% or MacConkey agar (purchased from Difco) were used. Plasmids and cassettes were maintained by the addition of ampicillin (100 µg/ml for K12, 200 µg/ml for EAEC), kanamycin (50 µg/ml for K12, 50 µg/ml for chromosomal insertion on EAEC, 100 µg/ml for plasmid-bearing EAEC), or chloramphenicol (40 µg/ml). Bromo-chloro-indolyl-β-D-galactopyranoside (X-Gal), Iso-propyl-thio-galactopyranoside (IPTG) and 2,2′-dipyridyl were purchased from Fluka. RNA polymerase holoenzyme (saturated with σ^70^) was purchased from Epicentre Biotechnologies. Custom oligonucleotides were synthesized by Eurogentec. With the exception of the Pfu Turbo Taq polymerase (Stratagen), restrictions and modification enzymes were purchased from New England Biolabs.

### Strain constructions

Deletion of the *lacZ* gene into the wild-type EAEC 17-2 strain was performed using the modified one-step inactivation procedure [48] with the pKOBEG plasmid [49] and oligonucleotides carrying 50-nucleotide extensions homologous to regions adjacent to the target gene (oligonucleotides are listed in Table S1). White colonies were screened on kanamycin LB plates supplemented with X-Gal (40 µg/ml) and IPTG (100 µM). The kanamycin cassette was then excised using plasmid pCP20. Deletion of the *pcnB* gene was performed into the 17-2Δ*lacZ* strain using the same procedure. The corresponding strain, 17-2Δ*lacZ*Δ*pcnB*, is considered as the WT reporter strain throughout the study. Deletions of the *fur* and of the *aggR* genes were done similarly into the reporter strain, whereas deletion of the *dam* gene was done into the reporter strain and its Δ*fur* derivative (oligonucleotides listed in Table S1).

### Plasmid constructions

Plasmids used in this study are listed in Table S1. Plasmids pKOBEG [49] and pCP20 [48] were provided by Arlette Darfeuille-Michaud and Barry Wanner respectively. Plasmid pBT4-1 [50], carrying the *E. coli* K12 *fur* gene was kindly provided by Sam Dukan and Maialene Chabalier through the Danièle Touati's strain collection. Plasmid pNKBOR [32] was kindky provided by Daniel Vinella. Plasmids encoding the transcriptional fusions were constructed by cloning the promoters from the *sci1* and *sci2* T6SS gene clusters (amplified by PCR using the Pfu Turbo [Stratagene] polymerase and corresponding oligonucleotides; *sci1*, from –578 to +18 relative to the initiation start codon of *sciH* (EC042_4524) [nucleotides 4852298-4852892; 596-bp]; *sci2*, from –421 to + 46 relative to the initiation start codon of *aaiA* (EC042_4562) [nucleotides 4892656-4893121; 467-bp]) upstream the '*lacZ* gene into the blunt *Sma*I site of the dephosphorylated pGE593 vector [30]. In these constructions, *lacZ* is under the control of the promoter of the corresponding gene. pT7.5 [51] derivatives carrying the *sci1*, *sci2*, and *cirA* promoters were engineered by a double PCR technique, allowing amplification of the DNA sequence of interest flanked by extensions annealing to the target vector. The product of the first PCR was then used as oligonucleotides for a second PCR using the target vector as template [4], [52]. pT7.5 derivatives carrying the *fur1* and *fur2* boxes of the *sci1* promoter were obtained by insertion using insertion quick change mutagenesis and complementary pairs of oligonucleotides. All constructs have been verified by restriction analyses and DNA sequencing (Genome Express).

### Beta-galactosidase assay

β-galactosidase activity was measured on whole cells by the method of Miller [55]. Reported values represent the average of at least three independent triplicates with a variation of less than 10% from the mean (standard deviation shown on graphics).

### Random mutagenesis using pNKBOR plasposon

The EAECΔ*lacZ*Δ*pcnB* strain carrying the reporter fusion plasmid pGE593-*sci1* was randomly mutagenized using the pNKBOR plasposon [32]. 350 ng of pNKBOR were transformed into EAEC Δ*lacZ*Δ*pcnB* electro-competent cells and the cell suspension was spread on fifty 20-cm LB agar plates (pH 8.0) supplemented with 100 µg/ml of 5-bromo-4-chloro-3-indolyl-β-D-galactopyranoside (X-Gal) and 50 µg/ml of kanamycine. Blue colonies were picked and streaked on identical plates. After validation, chromosomal DNAs were purified from overnight culture using the DNeasy Blood and Tissue kit (Qiagen), and digested by BglII. Upon ligation (T4 DNA ligase, Promega), fragments were transformed into CC118λpir electro-competent cells and spread on LB agar plates supplemented with kanamycine. Plasmids carrying the pNKBOR insert were extracted, verified by restriction analyses, and the chromosome-pNKBOR junction site was sequenced.

### *In vivo* Fur binding assay: Fur titration assay (FURTA)

The Fur titration assay relies on the activity of the *fhuF::lacZ* chromosomal transcriptional fusion in presence of a sequence carried on a multicopy plasmid [36]. In absence of plasmid or in presence of the pT7.5 vector, Fur represses the *fhuF::lacZ* fusion, leading to a *lac^−^* phenotype. The presence of a Fur binding site on the multicopy plasmid leads to a de-repression of the *fhuF*::*lacZ* transcriptional fusion, and a *lac^+^* phenotype. Briefly, 5 µl of an exponential culture of the H1717 strain bearing the pT7.5 vector or derivatives were spotted on a MacConkey plate supplemented with ampicillin and FeS0_4_ 30 µM. Controls to verify that the *fhuF::lacZ* fusion was active and responsive to iron levels in all the strains tested were done in parallel by spotting the same cultures on MacConkey plates supplemented with ampicillin (iron starved medium leading to de-repression [i.e., *lac^+^* phenotype] in all cases).

### Fur purification

This purification procedure is based on the ability of the native, non-recombinant Fur protein to bind metal affinity beads. The *E. coli* K12 Fur proteins (100% identical to the EAEC Fur protein) was purified by ion-metal affinity chromatography from DH5α carrying the pBT4-1 plasmid after induction with IPTG (200 µM) for 3 hours at 37°C as previously described [50]. 10^11^ cells were harvested, resuspended in buffer A (Tris-HCl 20 mM, NaCl 100 mM, Imidazole 5 mM, pH 8.0) supplemented with MgCl_2_ 10 mM, DNase 100 µg/ml and RNase 100 µg/ml, and disrupted by French Press. The cleared cell lysate containing the soluble fraction was loaded on a cobalt column (Talon, Clontech) equilibrated with buffer A, and after extensive washing with buffer A, the Fur protein was eluted in buffer A supplemented with 400 mM imidazole. Fractions were pooled, dialysed against buffer B (Tris-HCl 10 mM, NaCl 100 mM, pH 7.2) and 10-fold concentrated using the Amicon technology (Millipore, cut-off of 3,000 Da) before storage at -80°C. The final concentration of the Fur protein was 1.30 mg/ml.

### Electrophoretic mobility shift assay—EMSA

PCR products were generated using a mix of dNTPs supplemented with [α-^32^P]dGTP (5 µCi per PCR in a total volume of 50 µl; Perkin-Elmer), and purified using the Wizard Gel and PCR clean-up kit (Promega). Dam methylated substrates were prepared as described below except that the column-purified PCR products were digested by MboI (to hydrolyze un-methylated substrates) and full-length fragments were gel-purified using the Wizard gel and PCR clean-up kit (Promega). EMSAs with Fur and RNA polymerase holo-enzyme (RNAP) were adapted from previously published protocols [53], [54]. PCR products were incubated in a final volume of 10 µl in Fur EMSA binding buffer (10 mM Tris-borate, 40 mM KCl, 5% glycerol, 1 mM MgCl_2_, 100 µM MnCl_2_, 2 mM DTT, BSA 100 µg/ml and sonicated salmon sperm DNA 1 µg/ml, pH 7.5) at the concentration of 2 nM with increasing concentrations of Fur, of RNA polymerase holo-enzyme (saturated in σ^70^, Epicentre Biotechnologies), or of both Fur and RNAP. In competition experiments, Fur was added 5 min prior to RNAP addition. The mixture was incubated for 30 minutes at 25°C and then loaded on a pre-run 8% non denaturing polyacrylamide (Tris-borate) gel, and DNA and DNA-complexes were separated at 100 V in Tris-Borate buffer (45 mM Tris base, 45 mM boric acid, 100 µM MnCl_2_ buffer). Gels were fixed in 10% trichloro-acetic acid for 10 minutes, and exposed to Kodak BioMax MR films.

### *In vitro* Dam methylation and endonuclease restriction assays

The *in vitro* methylation protection assay was done as previously published [38] with modifications. Briefly, purified radio-labelled PCR products were *in vitro* methylated by the Dam methylase (New England Biolabs) in methylation buffer (50 mM Tris-HCl pH 7.5, 5 mM dithiothreitol (DTT), 5% glycerol, 20 mM KCl, 1 mM MgCl_2_, 100 µM MnCl_2_, bovine serum albumine (BSA) 100 µg/ml, in presence of 80 µM of S-adenosylmethionine (SAM)), as recommended by the manufacturer at 37°C for 4 hours. For competition experiments, the PCR products were first incubated with purified the Fur protein in methylation buffer for 30 min at 37°C before addition of the Dam methylase. Half of the mixture was loaded on an acrylamide gel to verify Fur binding by EMSA. The remaining was treated for 20 min at 65°C to heat-inactivate the Dam methylase, and column-purified. 5 nmoles of the PCR products were then digested by the indicated restriction endonucleases (all purchased from New England Biolabs) in the buffers recommended by the manufacturer. DNA fragments were resolved on a pre-run denaturing 12%-acrylamide gel at 200 V in TBE buffer. Gels were fixed in 10% trichloro-acetic acid for 10 minutes, and exposed to Kodak BioMax MR films.

### *In vivo* Dam methylation assay

The promoter-fusion vector pGE593-*sci1* was extracted from 5×10^9^ exponentially-growing cells from various backgrounds (wild-type, Δ*dam*, Δ*fur*) treated or not with 200 µM 2,2′-di-pyridyl using the Wizard Miniprep kit (Promega). Plasmids were digested by EcoRI and BamHI and the 600-bp promoter fragments were gel-purified (Wizard Gel and PCR clean-up kit, Promega). 200 ng of the fragment were then subjected to digestion by the indicated restriction endonucleases in the buffers recommended by the manufacturer. DNA fragments were resolved on a pre-run denaturing 12%-acrylamide gel at 200 V in TBE buffer and visualized under ultra-violet after GelRed staining as recommended by the manufacturer (FluoProbes).

## Supporting Information

Figure S1 Schematic representation of the *sci1* promoter region. The position of the *fur1* and *fur2* boxes and of the GATC sites are indicated (GATC-dis, distal GATC). Each GATC is part of a palindrome sequence recognized by specific methylation-sensitive (underlined name), methylation-insensitive (plain name) or methylation-dependent (italicized name) restriction enzymes. The size of the digestion products obtained for each enzyme (if accessible for digest) is indicated. Please note that Hpy188I has a palindromic penta-nucleotide recognition sequence, and therefore is only sensitive to methylation of top strand.
(PPT)

Table S1 Strains, plasmids, and oligonucleotides used in this study. ^a^ sequence adjacent to the target gene underlined, sequence annealing on pKD4 italicized. ^b^ consensus sequence underlined. ^c^ sequence complementary to target vector underlined. ^d^ sequence annealing on the target vector underlined. ^e^ sequence complementary to B oligonucleotide italicized.
(PDF)
