# Supplementary material for: An Epigenetic Switch Involving Overlapping Fur and DNA Methylation Optimizes Expression of a Type VI Secretion Gene Cluster
Source: PLoS Genet. 2011 Jul 28;7(7):e1002205. doi: 10.1371/journal.pgen.1002205 (PMC3145626; doi:10.1371/journal.pgen.1002205)
Supplement: Figure S1 — Schematic representation of the sci1 promoter region. The position of the fur1 and fur2 boxes and of the GATC sites are indicated (GATC-dis, distal GATC). Each GATC is part of a palindrome sequence recognized by specific methylation-sensitive (underlined name), methylation-insensitive (plain name) or methylation-dependent (italicized name) restriction enzymes. The size of the digestion products obtained for each enzyme (if accessible for digest) is indicated. Please note that Hpy188I has a palindromic penta-nucleotide recognition sequence, and therefore is only sensitive to methylation of top strand. (PPT) [file pgen.1002205.s001.ppt]

## Slide 1
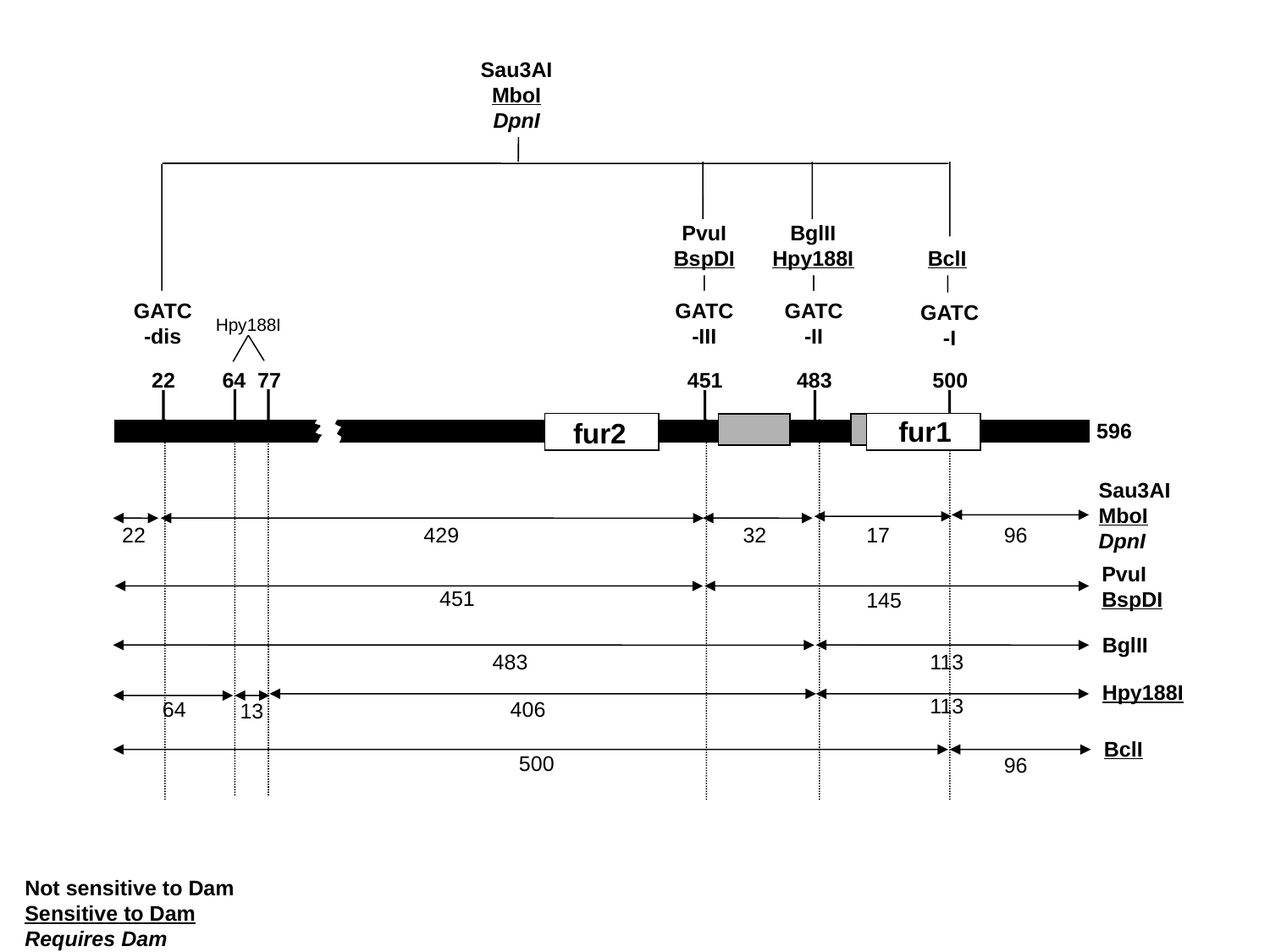

Sau3AI MboI DpnI
PvuI BspDI
BglII Hpy188I
BclI
GATC-dis
GATC-III
GATC-II
GATC-I
Hpy188I
22
64
77
451
483
500
fur1
fur2
596
Sau3AI MboI DpnI
22
429
32
17
96
PvuI BspDI
451
145
BglII
483
113
Hpy188I
113
64
406
13
BclI
500
96
Not sensitive to Dam Sensitive to Dam Requires Dam
