# Supplementary material for: An Epigenetic Switch Involving Overlapping Fur and DNA Methylation Optimizes Expression of a Type VI Secretion Gene Cluster
Source: PLoS Genet. 2011 Jul 28;7(7):e1002205. doi: 10.1371/journal.pgen.1002205 (PMC3145626; doi:10.1371/journal.pgen.1002205)
Supplement: Table S1 — Strains, plasmids, and oligonucleotides used in this study. a sequence adjacent to the target gene underlined, sequence annealing on pKD4 italicized. b consensus sequence underlined. c sequence complementary to target vector underlined. d sequence annealing on the target vector underlined. e sequence complementary to B oligonucleotide italicized. (PDF) [file pgen.1002205.s002.pdf]

Supplementary Table S1. Strains, plasmids and oligonucleotides used in this study.

Strain	description, relevant genotype	source
17-2	Wild-type enteroaggregative *Escherichia coli*	Arlette Darfeuille-Michaud
17-2Δ*lacZ*	17-2 deleted of the *lacZ* gene	This study
17-2Δ*lacZ*Δ*pcnB*	17-2Δ*lacZ* deleted of the *pcnB* gene	This study
17-2Δ*lacZ*Δ*pcnB*Δ*fur*	17-2Δ*lacZ*Δ*pcnB* deleted of the *fur* gene	This study
17-2Δ*lacZ*Δ*pcnB*Δ*aggR*	17-2Δ*lacZ*Δ*pcnB* deleted of the *aggR* gene	This study
17-2Δ*lacZ*Δ*pcnB*Δ*dam*	17-2Δ*lacZ*Δ*pcnB* deleted of the *dam* gene	This study
17-2Δ*lacZ*Δ*pcnB*Δ*fur*Δ*dam*	17-2Δ*lacZ*Δ*pcnB*Δ*fur* deleted of the *dam* gene	This study
BW25113	*E. coli* K12, Δ*(araD-araB)*, Δ*lacZ4787*	Datsenko and Wanner, 2000
BW25113 *fur*Ωkan	BW25113 *fur* insertional mutant (KEIO collection)	Baba *et al.*, 2006
BW25113 *dam*Ωkan	BW25113 *dam* insertional mutant (KEIO collection)	Baba *et al.*, 2006
MC4100	F-, Δ(*argF-lac*)U169, *araD139*, *rpsL150*, *relA1*, *deoC*, *rbs*, *flbB5301*	Laboratory collection
H1717	FURTA-reporter strain, MC4100 *aroB*, *fhuF*::λ*lac*Mu	Stojiljkovic and Hantke, 1994

Plasmids

pGE593	*lacZ* transcriptional reporter fusion cloning vector, Amp^R	Eraso and Weinstock, 1992
p*sci1*::*lacZ*	*sci-1* promoter cloned into *SmaI* site of pGE593	This study
p*sci2*::*lacZ*	*sci-2* promoter cloned into *SmaI* site of pGE593	This study
p*cir*::*lacZ*	*cirA* promoter cloned into *SmaI* site of pGE593	This study
pBT4-1	Fur production vector, Amp^R	Tardat and Touati, 1993
pNKBOR	Suicide vector , Tn, Kan^R	Rossignol et al., 2001
pKOBEG	Recombination vector, phage λ *recγβα* operon under the control of the pBAD promoter, Cm^R	Chaveroche et al., 2000
pT7-5	Cloning vector, T7 promoter, Amp^R	Tabor and Richardson, 1985
pT7-*sci1*	*sci-1* promoter cloned into pT7.5	This study
pT7-*sci2*	*sci-2* promoter cloned into pT7.5	This study

pT7-*fur1*	*fur1* box cloned into pT7.5	This study
pT7-*fur2*	*fur2* box cloned into pT7.5	This study
pT7-*cir*	*cirA* promoter cloned into pT7.5	This study

Oligonucleotides

For gene deletion[a]

Δ*lacZ*-5	GTTGTGTGGAATTGTGAGCGGATAACAATTTCACACAGGATACAGCTATG*TGTGTAGGCTGGAGCTGCTTCG*
Δ*lacZ*-3	TTTGTGTTTTTAAATAGTACATAATGGATTTCCTTACGCGAAATACGGG*CATATGAATATCCTCCTTAGTTC*
Δ*pcnB*-5	GCGTCATGCTGAGCTATGATTAGCCGCTATTTTTTTGTCCTGAATGATGT*TGTGTAGGCTGGAGCTGCTTCG*
Δ*pcnB*-3	CTGCTCCAGCGGAGAGGCCAGATTGCTGCCTATGGCAATATACGCCACTG*CATATGAATATCCTCCTTAGTTC*
Δ*fur*-5	TGTCACTTCTCGTAATGAAGTGAACCGCTTAGTAACAGGACAGATTCCGC*TGTGTAGGCTGGAGCTGCTTCG*
Δ*fur*-3	CGTGTATAAAAAAGCCAACCCGCAGGTTGGCTTTTCTCGTTCCGGCTGGC*CATATGAATATCCTCCTTAGTTCC*
Δ*aggR*-5	ACTCTAACGCAGAGTTGCCTGATAAAGACATTTTTTCATGTGAGAATGAT*TGTGTAGGCTGGAGCTGCTTCG*
Δ*aggR*-3	CATCACCAATATGTTTATAGCAATCTCAAATAATGATATAAAACATATTT*CATATGAATATCCTCCTTAGTTCC*
Δ*dam*-5	GCTGTCGGAGCTTTCTCCACAGCCGGAGAAGGTGTAATTAGTTAGTCAGC*TGTGTAGGCTGGAGCTGCTTCG*
Δ*dam*-3	CAGAATTGAGGGGGCAATCAAATACTGTTTCATCCGCTTCTCCTTGAGAA*CATATGAATATCCTCCTTAGTTC*

For promoter cloning into pGE593.

Prom*sci1*-5	CGCACCATGATCGTCTCTGTATCGC
Prom*sci1*-3	CTGAAACGAACTGCTCATGGCTCTCTC
Prom*sci2*-5	CTGGGGATTGGTGTTATGATCGGC
Prom*sci2*-3	CTCGTGGGGGGCGGGTTTTATC
Prom*cir*-5	GGCAACCAGACGGTATACCTGTGGC
Prom*cir*-3	GCGTACGGCAGCATCGCGGTG

For competition in gel shift experiments[b].

Fur-compet-up TATGCCGGATAATGATAATCATTATCTATGCCG
Fur-compet-bot CGGCATAGATAATGATTATCATTATCCGGCATA
RpoN-compet-up TATGCCGAAGGGTGGCACGATGATTGCATATGCCG
RpoN-compet-bot CGGCATATGCAATCATCGTGCCACCCTTCGGCATA

Sequencing oligonucleotides.

for sequencing chromosomal insertion using pNKBOR.
seq-NKBOR CAGAGATTTTGAGACACAACGTGGCTTTG

for sequencing *lacZ* fusions.
seq-lacZ-fus GTTGGGAAGGGCGATCGGTGCGG

for sequencing pT7.5 insertions.
seq-T7 GACAGCTTATCATCGATAAGCTTGGGCTGC

For cloning into pT7.5 vector and gel shifts.
using the double PCR technique[c]
Prom*sci1*-pT7-5 CTCACTATAGGGAGACCGGAATTCGAGCTCACGGTGACAAGCGCACCATGATC
Prom*sci1*-pT7-3 GGTCGACTCTAGAGGATCCCCGGGCTGAAACGAACTGCTCATGGCTCTCTCC
Prom*sci2*-pT7-5 CTCACTATAGGGAGACCGGAATTCGAGCTCTGGGGATTGGTGTTATGATCGGCATC
Prom*sci2*-pT7-3 GGTCGACTCTAGAGGATCCCCGGGCTCGTGGGGGGCGGGTTTTATCAAG
Prom*cir*-pT7-5 CTCACTATAGGGAGACCGGAATTCGAGCTC GGCAACCAGACGGTATACCTGTGGCTG
Prom*cir*-pT7-3 GGTCGACTCTAGAGGATCCCCGGGCGTACGGCAGCATCGCGGTG

using site-directed mutagenesis[d, e]
Afur *GCTCGAATTCCGGTCTCCCTA*TAGTGAGTCGTATTAATTTCG
Bfur1 *TAGGGAGACCGGAATTCGAGCTC*AGTCCTGATTATTTGCATTATATCGCCCGGGGATCCTCTAGAGTCGACC
Bfur2 *TAGGGAGACCGGAATTCGAGCTC*TCCTATAATGATCAAAATTAAATCAGCCCGGGGATCCTCTAGAGTCGACC
